# Functional interplay between long non-coding RNAs and Breast CSCs

**DOI:** 10.1186/s12935-022-02653-4

**Published:** 2022-07-21

**Authors:** Bashdar Mahmud Hussen, Ramiar Kamal Kheder, Sara Tharwat Abdullah, Hazha Jamal Hidayat, Heshu Sulaiman Rahman, Abbas Salihi, Mohammad Taheri, Soudeh Ghafouri-Fard

**Affiliations:** 1 Department of Pharmacognosy, College of Pharmacy, Hawler Medical University, Erbil , Kurdistan Region Iraq; 2 Center of Research and Strategic Studies, Lebanese French University, Erbil, Iraq; 3 Department of Medical Analysis, Faculty of Science, Tishk International University, Erbil, Iraq; 4 Medical Laboratory Science, College of Science, University of Raparin, Rania, KGR Iraq; 5 Department of Pharmacology and Toxicology, College of Pharmacy, Hawler Medical University, Erbil, Iraq; 6 Department of Biology, College of Education, Salahaddin University-Erbil, Erbil, Kurdistan Region Iraq; 7 Department of Physiology, College of Medicine, University of Sulaimani, Sulaimaniyah, Republic of Iraq; 8 Department of Medical Laboratory Sciences, Komar University of Science and Technology, Sulaimaniyah, Republic of Iraq; 9 Department of Biology, College of Science, Salahaddin University-Erbil, Erbil, Kurdistan Region Iraq; 10 Urology and Nephrology Research Center, Shahid Beheshti University of Medical Sciences, Tehran, Iran; 11 Institute of Human Genetics, Jena University Hospital, Jena, Germany; 12 Department of Medical Genetics, School of Medicine, Shahid Beheshti University of Medical Sciences, Tehran, Iran

**Keywords:** Breast cancer (BC), Cancer stem cells (CSCs), Breast CSC (BrCSC), lncRNAs, Therapeutic resistance

## Abstract

Breast cancer (BC) represents aggressive cancer affecting most women’s lives globally. Metastasis and recurrence are the two most common factors in a breast cancer patient's poor prognosis. Cancer stem cells (CSCs) are tumor cells that are able to self-renew and differentiate, which is a significant factor in metastasis and recurrence of cancer. Long non-coding RNAs (lncRNAs) describe a group of RNAs that are longer than 200 nucleotides and do not have the ability to code for proteins. Some of these lncRNAs can be mainly produced in various tissues and tumor forms. In the development and spread of malignancies, lncRNAs have a significant role in influencing multiple signaling pathways positively or negatively, making them promise useful diagnostic and prognostic markers in treating the disease and guiding clinical therapy. However, it is not well known how the interaction of lncRNAs with CSCs will affect cancer development and progression.

Here, in this review, we attempt to summarize recent findings that focus on lncRNAs affect cancer stem cell self-renewal and differentiation in breast cancer development and progression, as well as the strategies and challenges for overcoming lncRNA's therapeutic resistance.

## Introduction

Breast cancer (BC) is the most frequently diagnosed cancer in women worldwide, and it is ranked as second in terms of cancer related death in women [1]. Advances in early diagnosis and therapy techniques have reduced BC death and to some extent, improved patient prognosis and survival rate [2]. However, the emergence of drug resistance in BC patients has decreased the success rate of systemic therapies [3]. Cancer stem cells (CSCs), also known as cancer-producing cells, are characterized by the unique feature of self-renewal and differentiation into tumor-propagating cells [4, 5]. Approximately CSCs make up about 0.01–2% of all tumor cells in the body, which play a critical role in cancer initiation, progression, apoptotic endurance, and therapeutic resistance [6, 7]. CSCs first described in 2003 by Visvader and Linderman, describing CSCs role in acute myeloid leukemia (AML) [8].

Breast cancer stem cells (BrCSCs) can be formed from differentiated mammary cells due to disease-related mutations. The specific origin of BrCSCs has been a contentious issue for decades, and it's unclear where they come from. Exposure to environmental stimuli such as radiation causes genetic defects in non-malignant somatic cells and causes dedifferentiation of non-malignant somatic cells. Microenvironmental stimuli also can trigger a malignant transformation of differentiated cells into BrCSCs. Removing CSCs from the body is seen as a novel approach for the complete elimination of cancerous tumors and their prevention from recurrence [9].

It is estimated that 90% of the human genome is translated into RNAs [10–12], and less than two % of these transcripts are used to produce proteins [13]. Noncoding RNAs (ncRNAs) are a class of genes that have limited or no ability to code for proteins and are linked to cellular functions and disease development. Based on their length, non-coding RNAs (ncRNAs) are classified into two main classes based on their sizes: long ncRNAs (lncRNAs) more than 200nt in length and small ncRNAs less than 200nt in length [14].

According to certain studies, CSCs' activity is intimately linked to the abnormal expression of lncRNAs in malignant tumors. For example, OCT4 [15], SOX2 [16], KLF4 [17], and other stem cell-related pathways are regulated by many lncRNAs, which in turn affect CSC activities [18]. Furthermore, the control of miR-34a by lncRNAs, which were involved in tumor growth, was already reported in several studies to modulate CSC-like features in a range of malignancies [19, 20]. Recently, cancer-related research on lncRNA and CSCs is gaining growing attention. This study provides a comprehensive analysis of the current research status on lncRNAs and BrCSCs, their mechanisms of action, and the contribution of lncRNAs to tumorigenesis in BC by regulating CSCs, which could be used as potential diagnostic biomarkers and therapeutic strategies. This review can motivate further research to validate the highlighted lncRNAs-CSCs role as potential diagnostic biomarkers and therapeutic strategies in BC patients.

## LncRNAs in cancer biology

The first regulatory non-coding RNAs (ncRNAs) were discovered in bacteria in the 1980s, and then in most eukaryotic organisms. A few long noncoding RNAs (lncRNAs) such as H19 and Xist (X-inactive specific transcript) were identified prior to the genomic era, but they remained exceptions until the early 2000s [21]. They were later discovered to have five distinct origins: (A) a protein-coding gene undergoes structural damage and is transformed into a lncRNA; (B) chromosomal rearrangement brings two non-transcribed regions together, resulting in a lncRNA with multiple exons; (C) retrotranspositional duplication of a noncoding gene produces either a functional retrogene or a nonfunctional retropseudogene, both without encoding proteins; (D) two tandem duplication events result in adjacent repeats within a noncoding RNA; and (E) transposon insertion results in a functional lncRNA [22].

The presence of 18,805 LncRNAs has been established by GENCODE (release 40 (GRCh38.p13)), with about 39% of them found between genes and referred to as long intergenic ncRNAs (lincRNAs) [23]. Intronic lncRNAs, overlapping, and antisense are among the others [24]. They control a wide range of gene functions at the transcriptional [25], post-transcriptional [26], and epigenetic levels [27] by interacting with mRNAs, miRNAs, genomic DNA, and proteins. Thus, lncRNAs play a role in gene expression regulation, histone modification, chromatin remodeling, transcription and post-transcription splicing regulation, and translation.

**Fig. 1:**
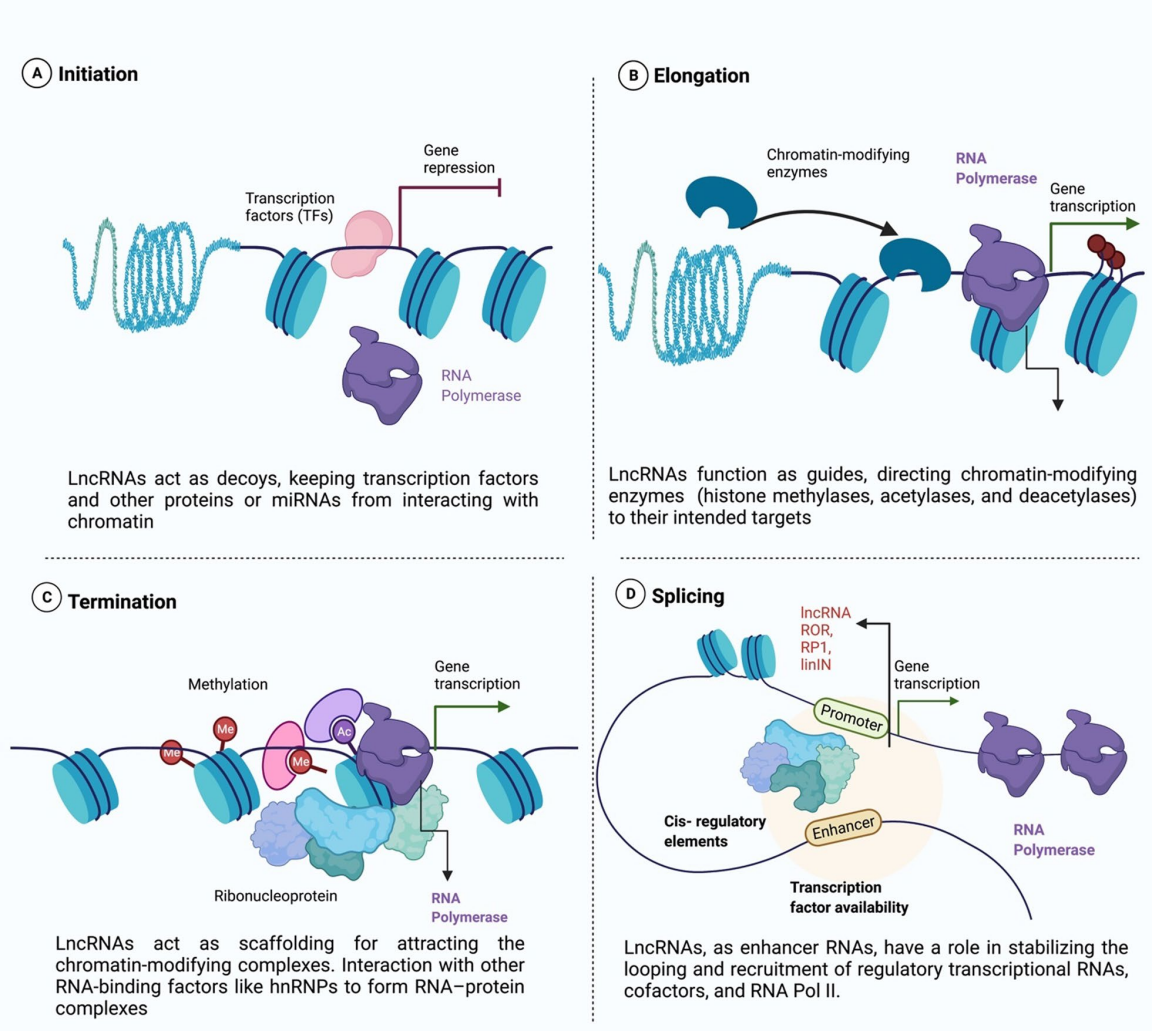
Transcriptional control via LncRNA

Their relation to tumorigenesis is dependent on abnormal expression in a variety of cancers, especially BC. Guide, signal, scaffold, and decoy are the main functions of lncRNAs [28] (Fig. 1). A number of lncRNAs can help regulate gene expression by directing the recruitment of transcriptional activators and suppressors to specific genomic locations [29, 30]. Guide lncRNAs are essential for the organization and localization of components at certain genomic loci in order to regulate the genome [31]. For example, HOTTIP, XIST, MEG3, COLDAIR, KCNQ1OT1, ANRIL, and TUG1 are all lncRNAs that can act as guides for epigenetic modifier recruitment to their specific loci [32]. Additionally, signal lncRNAs are expressed in response to external stimuli at a specified time and location within the cell. An example of this paradigm is that certain lncRNAs have regulatory functions, whereas others are essentially by-products of transcribing. Signaling lncRNAs have been shown to interact with chromatin-modifying enzymes such as histone methyltransferases to quiet their target genes by preventing their transcription or by forming heterochromatin [28]. Many lncRNAs may also function as scaffolding proteins, attracting chromatin remodeling complexes such as the PRC1 (polycomb repressive complex 1) and the PRC2 to inhibit specific target gene [33, 34]. In addition, several lncRNAs such as lncRNA ROR, DAPALR, and PANDA can serve as enhancer RNAs, have a role in stabilizing the looping and recruitment of regulatory transcriptional RNAs, cofactors, and RNA Pol II [35, 36]. LncRNA decoys inhibit transcription by preventing a specific effector from interacting with its intrinsic target [28]. By trapping regulatory components including miRNAs, altering chromatin subunits, and TF and restricting their availability, these lncRNAs indirectly regulate transcription process [37].

Since lncRNAs regulate gene expression, they have been connected to cellular mechanisms and diseases like stem cell maintenance and cancer metastasis [38, 39]. Perhaps this is because they can attach to DNA, RNA, or proteins.

## Long non-coding RNAs and BrCSC renewal

The ability to self-renew through mitotic cell division and the ability to differentiate into specialized cell types are two of the most fundamental characteristics of stem cells. They can produce more undifferentiated stem cells due to the latter potential. The latter feature, on the other hand, allows the development of differentiated cell types within an organ.

According to the CSC hypothesis, malignancies are caused by the transformation of stem or progenitor cells capable of multilineage differentiation [40]. Although CSCs make up a small proportion out of the total tumor mass, their survival is dependent on this small cell population [41].

These cells, which have been given the designation of CSCs, exhibit characteristics of both stem cells and cancer cells. Self-renewal, differentiation, and the ability to form tumors are some of the characteristics of these cells. Firstly, Lapidot et al. revealed that human AML is organized as a hierarchical structure that is derived from primitive hematopoietic cells [42]. A decade later, this paradigm was used to study BC in NOD-SCID mice by Al-Hajj et al., who uncovered a tumorigenic subpopulation in the mice [43]. Published studies in the following years have demonstrated that CSCs are present in a wide range of cancers [44]. Later, Rosen and Jordan, showed that the Wnt, Notch, and Hedgehog (Hh) signaling pathways, which drive stem cell self-renewal can also control the functions of CSCs [45].

As cancer progresses, several factors are hypothesized to influence the characteristics of CSCs such as lncRNAs. Interestingly, lncRNAs have the potential to influence the CSCs to self-renew by modulating key cancer-related pathways through overexpression, deficiency, or mutation. These pathways include the epithelial-mesenchymal transition (EMT) [46], the Notch pathway [47], the Rho-GTPase system [48], and the NF-B cascade [49]. For example, EMT and BrCSC self-renewal has been shown to be induced by overexpression of linc00617, which decreases E-cadherin levels while elevating N-cadherin and Vimentin levels [50]. Furthermore, due to the enrichment of the CSC fraction, BC cell populations that overexpress lincRNA are more likely to form mammospheres and become tumorigenic. It binds to the promoter of the Sox2 gene and activates its transcription by recruiting hnRNP-K to the promoter which promotes self-renewal ability of CSCs [51]. Figure 1 describes the roles of lncRNAs in BC cells that can either promote or inhibit gene expression.

## Prognostic role of LncRNAs in BrCSCs

In stem cell biology, lncRNAs are emerging as key contributors. LncRNAs have been identified to modulate pluripotency and differentiation of embryonic stem cells (ESCs) and induced pluripotent stem cells (iPSCs), according to several studies. In addition, lncRNAs are becoming more significant adult stem cell regulators. OCT4, SOX2, KLF4, and PcG, all of which are critical in ESCs, have been found to be active in CSCs [52]. Depending on investigations on the existence and development of BC, BrCSCs appear to be resistant to chemotherapy, radiation, and hypoxia [53–55]. Additionally, the tumorigenicity and invasiveness of BrCSCs play a significant role in the initiation, proliferation, metastasis, and recurrence of BC [56].

In cancers dysregulation of lncRNAs [38, 57] contemplate the role of lncRNA in controlling stem cell signaling in cancer cells [58]. Further exploration of versatile functions of lncRNAs in CSC may unravel hidden and novel therapeutic strategies for overcoming chemotherapy resistance a major obstacle in BC treatment. LncRNAs, including MALAT-1, HOTAIR, and H19, are commonly found in patients with BC because they affect key signaling pathways involved in tumor promotion and suppression [38]. As a result of the research of deregulation mechanisms, it has become clear that lncRNAs might be used as biomarkers in the diagnosis, prognosis, and therapeutic strategies. Interestingly, lncRNAs play a pivotal role in BrCSCs by engaging in several critical pathways [59] and significantly impact BC progression. LncRNAs can promote or impede BC cell invasion and metastasis by activating or repressing the EMT during the initiation and progression of BC [60].

A variety of LncRNAs have been reported to be overexpressed in BrCSCs, including HOTAIR [61], MALATI [62–64], H19, DANCR [65–67], NR2F1-AS1 (NAS1) [68], NEAT1 [69, 70], NRAD1 [71], LINC-ROR [72, 73], linc00617 [50], CCAT1 [74], RP1-5O6.5 (RP1) [75], and lncRNA-Hh [76], and they all play a role in various cancer hallmarks, by the modulation of several proteins and miRNAs (Table 1).

**Table 1:** Summery on the role of lncRNAs as transcriptional gene regulators in BrCSCs

LncRNAs	Detection methods	Expression patterns in BC	Targeted gene	Mode of actions	Clinical features	Related Cancer Hallmarks	Refs
HOTTIP and CBR3-AS1	RT–qPCR	↑	CCND1	The expression of HOTTIP and CBR3-AS1significantly increased the CCND1	Poor prognosis and tumor grade	Sustaining proliferative signaling	[77]
PANDAR and PANTR1	RT–qPCR	↑	CDKN2C		Clinical stage	Sustaining proliferative signaling	[78]
MALAT1	RT–qPCR	↑	Slug, KDM5B, CD133, PD-L1, miR-1, miR-182-5p	-Suppressing miR-1 expression -Interaction with Slug, KDM5B, and enhancing BC progress -Forms a repressive complex with RPB HuR, which regulates CD133	Poor survival	Activating invasion and metastasis Resisting cell death	[62] [63] [64]
H19	RT–qPCR	↑	LIN28, PDK1, HIF-1α, LIN 28, miR-103, miR-107, let-7, miR-29b-1	-Sponging miRNA tumor suppressors -Glycolysis and BCSC maintenance are aided by increasing PDK1 expression	Tumor size, hormone negativity, and nodal status	Inducing angiogenesis Deregulating cellular enargites	[79] [66] [67]
DANCR	RT–qPCR	↑	EZH2, SOCS3	-Excessive expression of DANCR was associated with decreased SOCS3 expression via epigenetic regulation of EZH2 and the H3K27me3 signal-The expression of DANCR significantly increased NF‐κB and STAT3 activation	Lymph node metastasis or advanced tumor grades	Activating invasion and metastasis	[65]
NR2F1-AS1 (NAS1)	RT–qPCR	↑	NR2F1, Np63	-This inhibits Np63 transcription by interacting with NAS1 and recruiting the RNA-binding protein PTBP1	Metastatic dormancy	Activating invasion and metastasis	[68]
NEAT1	RT–qPCR	↑	HMGA2, miR-211	-Through the miR-211/HMGA2 axis, NEAT1 produced EMT and 5-FU resistance	Induced EMT and 5-FU resistance	Activating invasion and metastasis	[69]
NEAT1	RT–qPCR	↑	CD44 + /CD24-, ALDH + , SOX2 +	-Stem cell populations such as CD44 + /CD24, ALDH + , and SOX2 + are reduced by NEAT1 to induce drug resistance	Chemoresistance	Activating invasion and metastasis	[70]
NRAD1	ChIRP-seq	↑	ALDH1A3	-lncRNA with chromatin-binding properties that are controlled by ALDH1A3 and facilitates gene expression	Overall survival	Deregulating cellular enargites	[71]
LINC-ROR	RT–qPCR, NGS	↑	Nanog, Oct4, SOX2, MECP2, miR-145, miR‐194‐3p	-Affects the expression of Nanog, Oct4, and SOX2 and regulates the maintenance of hESCs via sponging miR-145 -Linc-ROR/miRNA-194-3p/MECP2 axis mediates the tumor progression and treatment sensitivity	Drug sensitivity	Activating invasion and metastasis	[72] [73]
LINC01133	RT–qPCR	↓	EZH2, SOX4	-EZH2 binding mediates SOX4 transcriptional suppression, which in turn reduces BC invasion and metastasis	Advanced TNM stage and lymph node metastasis	Activating invasion and metastasis	[80]
linc00617	RT–qPCR	↑	Sox2	-By stimulating the transcription of Sox2, it promotes BC growth and metastasis	Advanced tumor grade and lymph node metastasis	Activating invasion and metastasis	[50]
CCAT1	RT–qPCR	↑	ZFX, miR-218	-Using miR-218/ZFX, CCAT1 encourage the growth of BC	-	Activating invasion and metastasis	[74]
RP1-5O6.5 (RP1)	RT–qPCR	↑	P27kip1	-Represses P27kip1 translation, which aids in BC growth and metastasis	TNM stage, tumor grade, lymph node, and distant metastasis	Activating invasion and metastasis	[75]
lncRNA-Hh	RT–qPCR Microarray	↑	SOX2, OCT4	-Hh promotes GLI1 expression and stimulates the expression of SOX2 and OCT4	-	Sustaining proliferative signaling	[76]

### LncRNAs regulate BrCSCs through epigenetic modifications

There are several ways in which the DNA sequence is not altered but epigenetic regulation affects gene expression; they include histone modifications, methylation of DNA, and genomic imprinting [81, 82]. LncRNAs are essential modulators of the epigenetic state of the human genome (Fig. 1). Chromatin remodeling and modification complexes are attracted to particular places by certain lncRNAs [83]. Examples of lncRNAs that can influence carcinogenesis include EPB41L4A-AS2 [84], BLAT1 [85], BCLIN25 [86], ANRASSF1 [87], H19 [88], and piR-823 [89] which can control DNA methylation. Han and his group found that BLAT1 expression is controlled by lowering the degree of promoter DNA methylation of CpG islands [90]. They showed that cancer patients with tumors that are BLAT1-hypomethylated have a decreased chance of surviving long-term. The high BLAT1 expression with hypomethylation at CpG sites in BC may be associated with the aggressiveness of the disease. BCLIN25 reduces miR-125b expression and elevates BC risk by increasing CpG methylation at the miR-125b promoter region, increasing ERBB2 expression [86]. Calanca et al. found that the tumor suppressor RASSF1A's epigenetic suppression has been linked to the lncRNA ANRASSF1. They proved that ANRASSF1 also is a therapeutic target with the potential to restore the restrictive chromatin changes generated by PRC2 at the promoter of RASSF1A, potentially leading to upregulation of RASSF1A in BC [91]. In addition, a lncRNA known as H19 suppresses the maternal allele at the H19/IGF2 gene via the methylation process in BC, resulting in a more aggressive phenotype [88]. A recent study demonstrated that lncRNA piR-823 overexpression in luminal BC cells activated Wnt signaling and induced cancer cell stemness by increasing DNMTs (DNMT1, DNMT3A, DNMT3B) expression and promoting adenomatous polyposis coli (APC) methylation [89].

In addition, lncRNA decreases gene transcription by recruiting proteins that modify histones or remodel chromatin. Histone regulators interact with the lncRNA HOTAIR, which results in transcriptional gene suppression in chromatin dynamics [92]. For example, HOTAIR contributes to the silencing of miR-205 by altering the balance of histone modifications on the miR-205 promoter between H3K4me3 and H3K27me3, which controls the production of cyclin J (CCNJ) [93]. Furthermore, HOTAIR inhibits miR-7 expression, leading to increased SETDB1 expression in BC stem cells, inducing EMT [94]. Moreover, HOTAIR promotes EMT by acetylating histone H3K27 to methylate the E-cadherin promoter, inhibiting E-cadherin production [95].

One of the most well-studied lncRNAs is Xist, active in the early phases of X chromosomal inactivation in female embryos. First instances of lncRNA directly engaged in the production of repressive chromatin are found in Xist [96]. Furthermore, Xist through particular RNA sequences, organizes the anchoring of chromatin modifiers to one of the two X chromosomes, enabling transcriptional silencing [97].

Several noncoding RNA molecules function as ligands and form complexes with transcription factors to regulate gene transcription [98, 99]. cis and trans regulation are two mechanisms used by lncRNAs to control gene transcription. Classifying lncRNAs into four categories by Wang and Chang namely; (decoy molecules, guiding molecules, scaffolding molecules, and signal molecules) contributed significantly to the progress of the lncRNA research area in the past few years [100]. The involvement of lncRNAs as a decoy, guiding, scaffolding, and signal molecules at the transcriptional level promotes or suppresses gene expression in BC metastasis (Fig. 1). For example, lncRNAs Lethe, NORAD, and PANDA work as "decoys," mimicking and competing with miRNAs or proteins in the nucleus [28, 98, 101] (Fig. 1a). Furthermore, the “guiding lncRNAs,” such as HOTAIR [102] and lincRNA-p21 [103], may directly act on transcriptional factors or chromatin modifiers and protein complexes and attract them to find particular target gene(s) to alter the transcription process (Fig. 1b) [104]. Moreover, LncRNAs MALAT1 [105], HOTAIR [106], and LINP1 [107] can form ribonucleoprotein complexes and regulate gene expression as scaffold molecules (Fig. 1c). In addition, to limit the activities of regulatory miRNAs, they also function as decoy microRNA-binding sites, like ceRNAs [108]. Additionally, various studies have revealed that lncRNAs can be used as molecular signals since they are transcribed at very particular times and locations, allowing cells to integrate developmental clues, read the cellular environment, or respond to a variety of stimuli as chemical signals [100, 109] (Fig. 1d). According to Wang and his colleagues, the lncTCF7 promotes TCF7 expression by recruiting SWI/SNF to TCF7's promoter. These steps may cause Wnt signaling to be activated, which might lead to cancer stem cells self-renewing and tumor cell proliferation [110]. Besides, the specific targets of guide lncRNAs are promoted by RNA–RNA, RNA–protein, and RNA–DNA interactions (Table 2).

**Table 2:** LncRNAs regulate BrCSCs through ceRNAs mechanism

LncRNA	Sponging miRNAs	Targeted genes and their expressions	Signaling pathways	Findings	References
Linc-ROR	miR-145	↑ ARF6	Linc-ROR-miR-145	Increased growth of the mammosphere stem cell population	[118]
LncRNA H19	Let-7	↑ ESR1	LncRNA H19/Let-7 miRNA	Cancer development, as well as cell metabolism	[119]
HOTAIR	miR-34a	↑ SOX2	SOX2 signaling	BrCSC proliferating and self-renewal capacity	[131]
LUCAT1	miR-5582-3p	↑ TCF7L2, SOX2, ↑ β-catenin	LUCAT1-miRNA-5582-3p-TCF7L2	Regulates BC stemness	[123]
lncCCAT1	miR-204, miR-211, miR-148a, miR-152	TCF4, ↑ β- catenin, ↓ ANXA2	Wnt/β-catenin pathway	Promotes BrCSC proliferating, stemness, and migrating	[124]
SPRY4-IT1	miR-6882-3p	↑ TCF7L2	SPRY4-IT1/ miR-6882-3p	Promotes proliferation of BrCSCs	[125]
LincK	miR-200	↑ ZEB1	LincK/ ZEB1/miR-200	Contributes to breast tumorigenesis and EMT	[126]
LSINCT5	miR-30a	↑ TCF4, c-Myc	Wnt/β-catenin pathway	Increases proliferation, motility, and EMT	[127]
HOTTIP	miR-148a-3p	↑ WNT1	Wnt/β-catenin signaling	Correlated well with the progression of BC	[130]
LINC00511	miR-185-3p	↑ NANOG, E2F1	miR-185-3p/E2F1/Nanog signaling	Tumorigenesis and stemness	[128]
LINC01133	miR-199a	↑ KLF4, FOXP2	miR-199a-FOXP2 signaling	Stemness and growth	[129]
LncRNA ES1	miR-106b	↑ E-cadherin, SOX2, OCT4 miR-200, miR-306	Oct4/Sox2/MiR-302 signaling	Stimulates cell migration and EMT	[132]
SOX21-AS1	miR-429	↑ SOX2	SOX21-AS1/miR-429/SOX2 signaling	Tumor invasion, proliferation, and the expression of stem factors	[133]
FEZF1-AS1	miR-39a	↑ NANOG, OCT4, SOX2	FEZF1-AS1/miR-30a/Nanog signaling	Increases CD44 + /CD24-, mammosphere-forming capacity, stem factors, and stimulates tumor growth and metastasis	[134]
PDCD4-AS1	miR-10b-5p	↑ IQGAP2	PDCD4-AS1/ miR-10b-5p/ IQGAP2	Increases the expression of IQGAP2 via miR-10b-5p, which aids BC cell proliferation, invasion, and migration	(135)

### Regulation of transcription in BrCSCs by LncRNAs

**Fig. 2:**
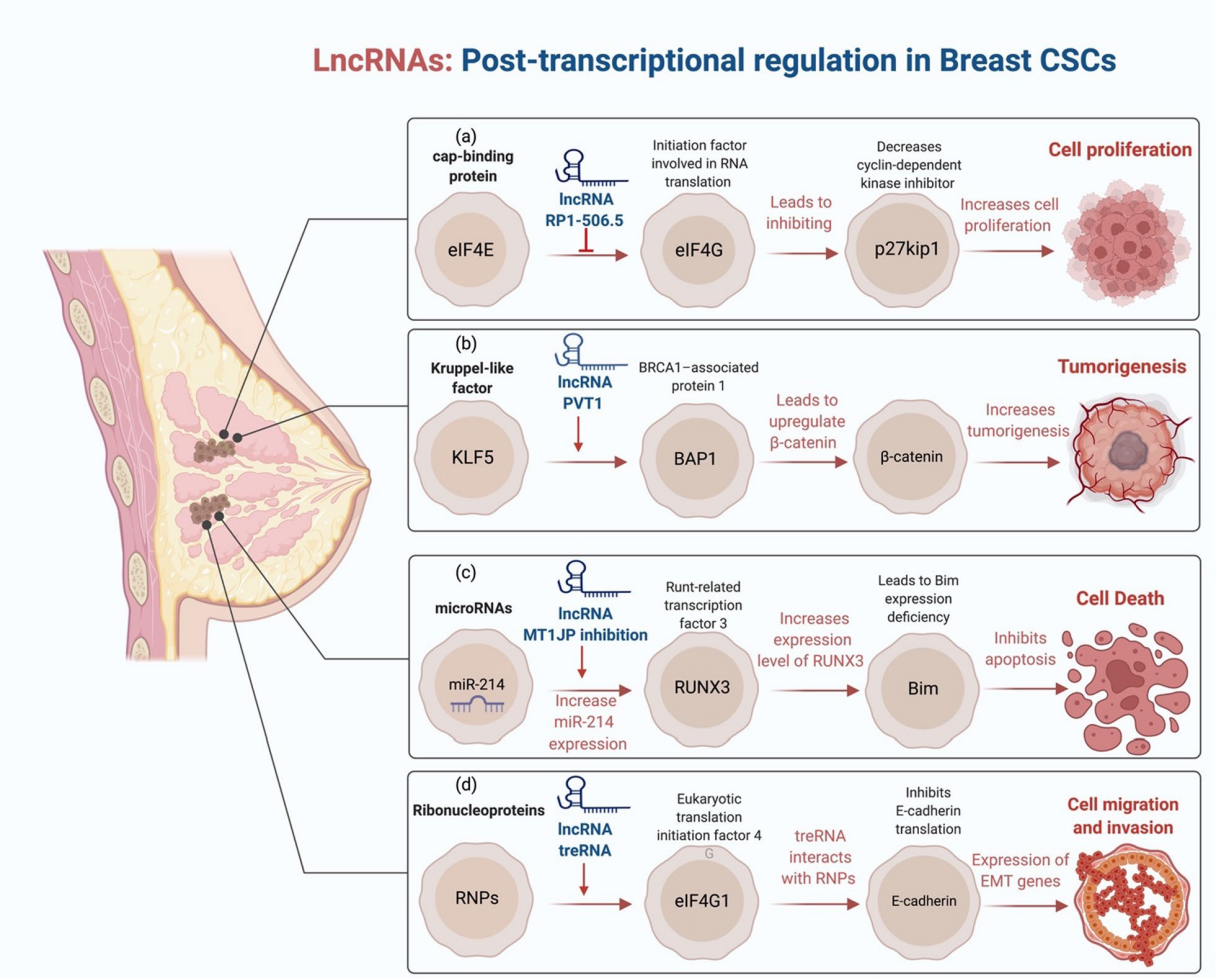
LncRNAs and Post-transcriptional regulation in BrCSCs. **a** LncRNAs inhibit the initiation factor that involved in RNA translation such as elF4G which decreases the level of cyclin-dependent kinase inhibitor and promote cell proliferation. **b** Due to lncRNA PVT1 binding to KLF5 and promoting its stability through BAP1, beta-catenin signaling is accelerated, resulting in an increase in the development of beta-catenin tumors in BC patients. **c** LncRNA MT1JP suppression enhances miRNA-214 gene transcription through altering the miRNA-214/RUNX3 Axis, which results in Bim expression defect and suppresses the apoptosis process. **d** lncRNA treRNA forms complexes with ribonucleoproteins (RNPs) that inhibit E-cadherin translation by targeting eIF4G1 and increase the expression of EMT genes, resulting in increased cell migration and invasion

LncRNAs influence the translation of mRNAs and regulate their integrity at the posttranscriptional stage by creating double-stranded RNA with mRNAs or via binding to proteins. Tang and his group found that proliferation, colony formation, and development of orthotopic xenograft tumors were all reduced by the depletion of PVT1. Through KLF5/beta-catenin signaling, they also showed that lncRNA PVT1 controls TNBC [111]. Antisense lncRNAs are the most common lncRNAs engaged in mRNA post-transcriptional regulation. There are various ways in which lncRNAs might alter the splicing process of pre-mRNA, either alone or with other splicing factors. For example, the lncRNA RP1-506.5 interacts with eIF4E and inhibits eIF4E from binding to eIF4G, inhibiting p27kip1 translation and adversely regulating Snail levels in BC cells [75]. Likewise, Beta-catenin signaling is increased due to lncRNA PVT1 binding to KLF5 and increasing its stability via BAP1, which increases BC tumorigenesis [111]. The biological processes of malignancies are influenced by lncRNA dysfunction, which has been linked to tumor prognosis. The lncRNA encoded by metallothionein 1 J (MT1JP) has been linked to carcinogenesis, and its expression is downregulated in different types of cancer [112–114]. Meanwhile, in vivo and in vitro studies recently showed that MT1JP inhibition increases miRNA-214 gene expressions in BC cells by modulating miRNA-214/RUNX3 Axis [115]. Moreover, it was also shown that lncRNA treRNA interacts with ribonucleoproteins (RNPs) (PUF60, SF3B3, FXR1, FXR2, PUF60, and hnRNP K) to produce a treRNA-RNP complex that inhibits E-cadherin translation by targeting eIF4G1 [116] (Fig. 2).

### LncRNAs regulate BrCSCs through ceRNAs mechanism

It has been found that RNAs can interact with each other in a novel way (ceRNA networks), and that these interactions are critical to the formation of cancers. They have the potential to act as therapeutic targets in addition to being diagnostic and prognostic markers. According to literature studies, numerous lncRNAs act as ceRNAs in BrCSCs, targeting miRNAs and altering the EMT process. For instance, linc-ROR affects the expression of the stemness factors Nanog, Oct4, and SOX2 and regulates the maintenance of human embryonic stem cells (hESCs) via sponging miR-145 [72]. Further, upregulation of linc-ROR in BC resulted in a higher stem cell phenotype and greater mammosphere development, indicating that the Linc-ROR-miR-145 pathway is critical in cancer stemness [117, 118]. In addition, one of the most well-known and investigated ceRNA mechanisms is the lncRNA H19/Let-7 miRNA [119]. BrCSCs and breast tumors had a significant level of lncRNA H19 expression. Increasing the amount of H19 in tumor cells permits them to survive treatments [120], also H19 is involved in controlling BrCSCs' division by sponging microRNA let-7 which provide potential strategies for stem cell invasion and mammosphere formation [121]. Likewise, lncRNA HOTAIR (homeobox antisense transcript antisense RNA) is associated with the growth and spread of BC cells [122]. BrCSC derived from MCF7 or MB231 have high levels of HOTAIR up-regulation, which affects BrCSC growth, migration, and self-renewal. HOTAIR is also known to influence miR-34a, which in turn promotes Sox2 expression, according to a recent study [61]. HOTAIR can reduce the expression of miR-7 by regulating HoxD10 in MCF-7 and MDA-MB-231 BrCSCs [94]. Additionally, LUCAT1 promotes BrCSC stemness by sponging miR-5582-3p and promoting TCF7L2 and the Wnt/β-catenin pathway. On the other hand, BrCSCs self-renewal was hindered by LUCAT1 downregulation, which enhanced miR-5582-3p expression [123]. Moreover, lncCCAT1 is highly expressed in BrCSCs and is linked to poor patient consequences. LncCCAT1 overexpression promotes BrCSC proliferation, stemness, migration, and invasion capabilities. Depends on the above results, lncRNAs can be used as a marker of the patient's prognosis and the risk of their tumor recurrence which may apply in clinical translation. Meanwhile, miR-204/211, miR-148a/152, and ANXA2 can all interact with LncCCAT1, which in turn can activate TCF4 or promote the translocation of β-catenin to the nucleus and activate Wnt signaling [124]. Additionally, in a recent study, Song and his team found that lncRNA SPRY4-IT1 enhances BC cell proliferation and stemness, as well as BrCSC renewal and stemness maintenance, via promoting the expression of TCF7L2 through targeting miR-6882-3p [125].

**Fig. 3:**
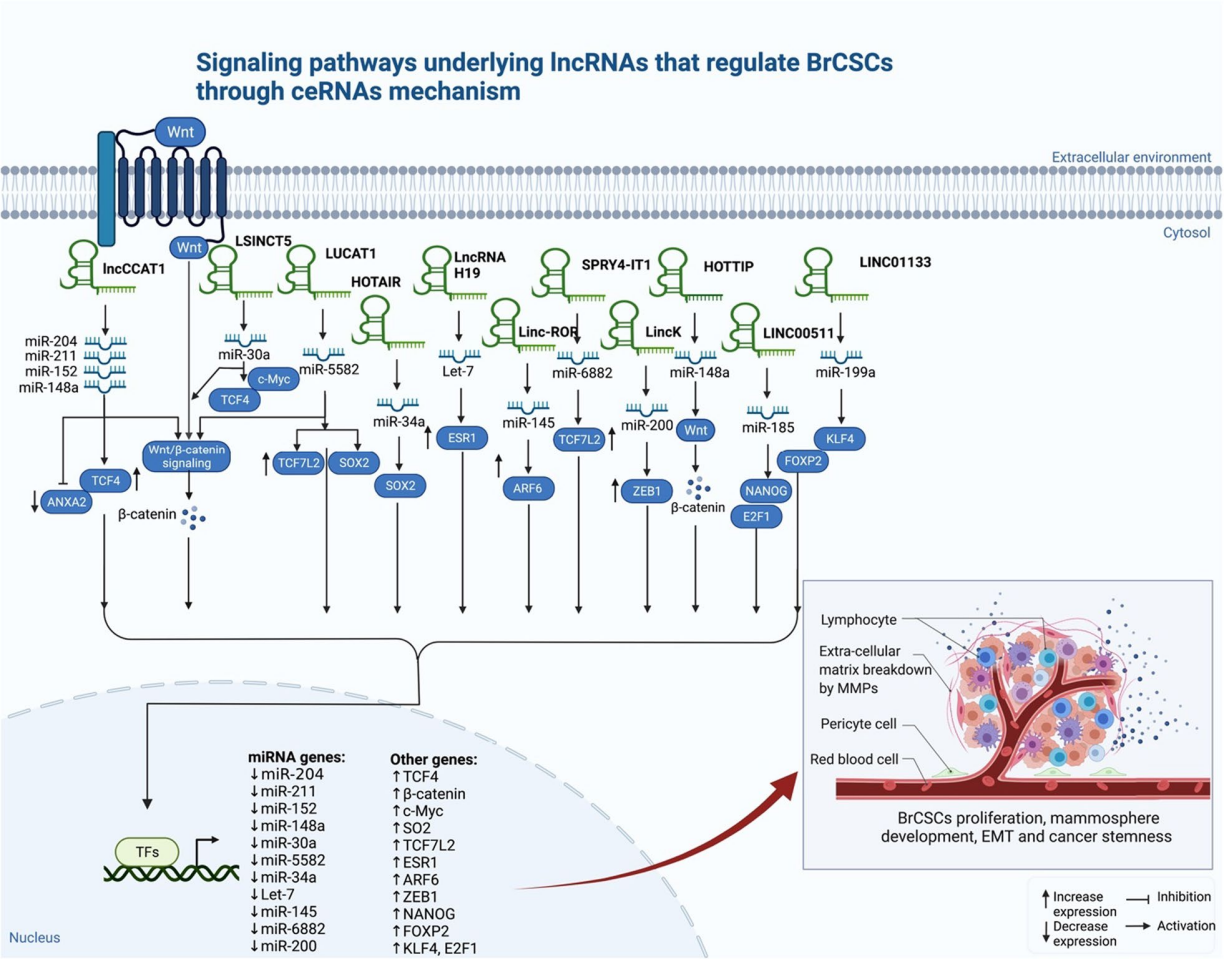
Different signaling pathways in which lncRNAs regulate BrCSCs through ceRNAs mechanism (miRNAs gene inhibition) which increases the mamosphere development, EMT and cancer stemness

Besides, LincK also controls ZEB1 through the sponging of miR-200, which contributes to breast tumorigenesis [126]. In a recent study, lncRNA LSINCT5 has increased cell motility by sponging miR-30a from the Wnt/β-catenin pathway. TCF4 and c-Myc expression was likewise downregulated in cells in which LSINCT5 was knocked out, resulting in decreased proliferation, motility, and EMT [127]. Furthermore, by promoting the miR-185-3p/E2F1/Nanog axis, lncRNA LINC00511 leads to BC tumorigenesis and stemness [128]. Similarly, In TNBC models, LINC01133 acts as a direct mediator of the MSC-triggered miR-199a-FOXP2 axis, enhancing phenotypic and growth characteristics of CSC-like cells [129]. Lastly, evidence supports that the lncRNA, HOTTIP (HOXA transcript at the distal tip), is upregulated in a variety of malignancies, including BC. HOTTIP is also involved in several biological activities, such as maintaining stem cell viability. As a molecular sponge for miR-148a-3p, HOTTIP controls the CSC-like features of BrCSCs, enhancing WNT1 transcription and providing a novel therapeutic target for BC patients [130] (Fig. 3).

Table 2 provides additional examples of lncRNA that regulate BrCSCs through ceRNAs mechanism.

Overall, in terms of lncRNA aberrant expression and the causative factors, lncRNAs may be useful prognostic biomarkers in BrCSCs. Additionally, lncRNAs have the potential to be targeted to reverse the process of carcinogenesis, making them valuable therapeutic targets for treatment of cancer.

Since most of lncRNAs/miRNAs axes (ceRNA axes) have been only assessed in one paper, there is no way to compare the results of studies. However, it is possible that a single lncRNA acts as a molecular sponge for more than one miRNA. In fact, it is possible to identify several lncRNAs/miRNAs axes in which some of the contributing ncRNAs have common roles.

## LncRNAs participate in different pathways for modulating BrCSCs

Because lncRNAs are highly abundant in BrCSCs, they can play a variety of regulatory functions. In recent years, the importance of lncRNAs has been recognized in BrCSCs, with their functional significance and clinical consequences being highlighted recently. Here, the regulatory functions of lncRNAs on BrCSCs are discussed in terms of their many modes of action.

### LncRNAs regulate BrCSCs through HIF signaling pathway

As it turns out, hypoxia influences a specific subset of long noncoding RNAs. Hypoxia-responsive lncRNAs (HRLs) such as NORAD, LncHIFCAR, AC020978, KB-1980E6.3, RAB11B-AS1 may have a role in cancer cell survival and disease progression [101, 136, 137]. HRLs may be classified into two categories: those dependent on hypoxia-inducible factors (HIF) and those not dependent on HIF. Specifically, the hypoxia response element (HRE), located in the promoter regions of HRLs, may directly bind to HIF in hypoxic tumor microenvironments and control their expression by modulating their transcription. Conversely, the HRLs that are excessively expressed may trigger a tumor-specific molecular profile and contribute to the development of tumor characteristics [138]. The molecular properties of HRLs in BC, however, remain unsolved.

Recently, in an in vitro and in vivo studies undertaken in a hypoxic condition, Zhu and colleagues found that the KB-1980E6.3 enhanced BrCSC self-renewal and tumorigenesis [139].

They observed that HIF-1α might interact directly with the HRE in the KB-1980E6.3 promoter to control its transcription activities in hypoxia conditions. Furthermore, Because of its capacity to bind to IGF2BP1, lncRNA KB-1980E6.3 was critical for BrCSC stemness because it increases the stability of c-Myc mRNA and enhances it BC cells' responsiveness to hypoxia [139].

### LncRNAs regulate BrCSCs through Hh, Wnt, Hippo signaling pathways

**Fig. 4:**
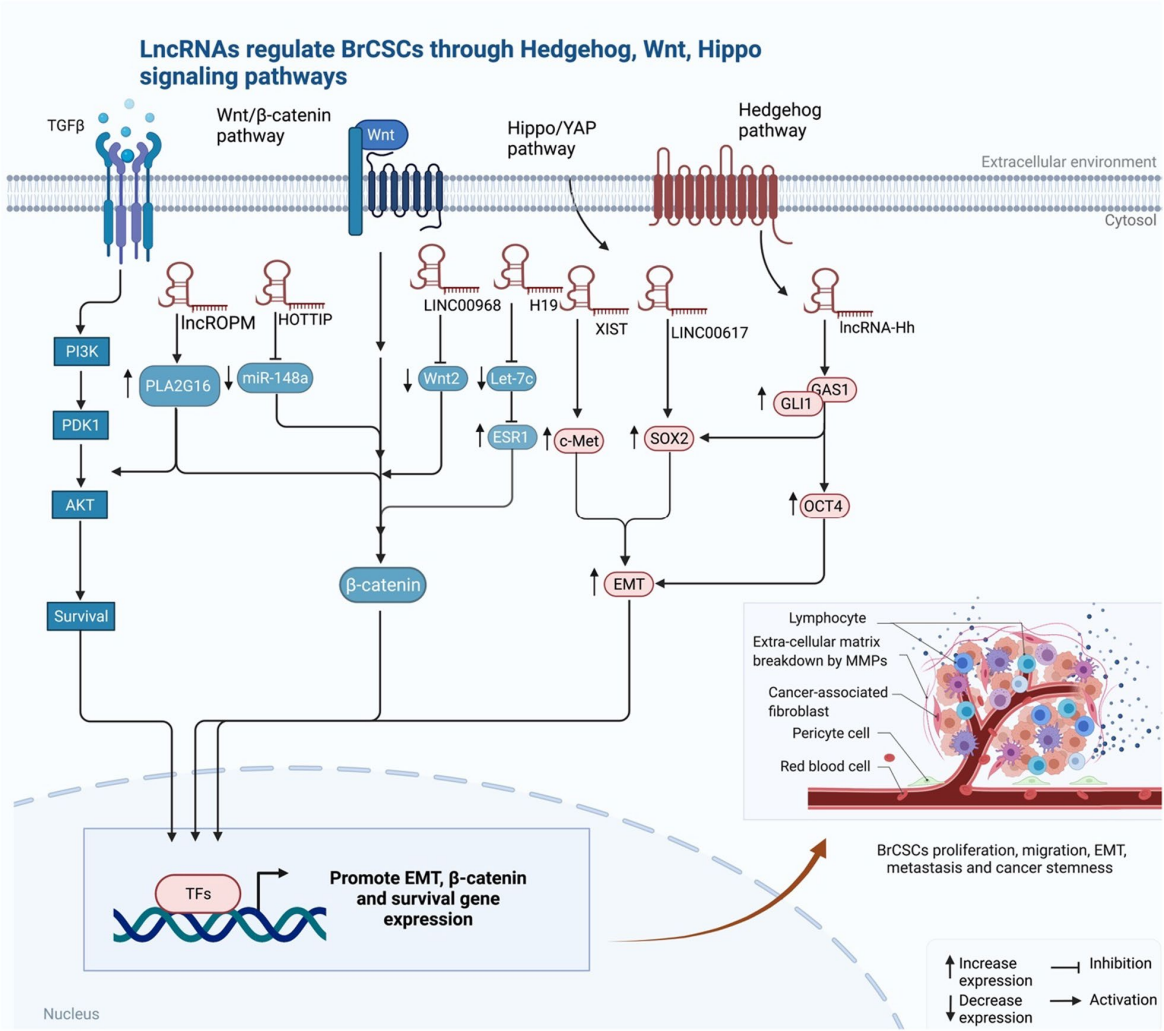
LncRNAs regulate BrCSCs by promoting EMT, β-catenin, and survival genes, which enhances proliferation, EMT, metastasis, and cancer stemness

The estrogen receptor (ER) regulates the Hh, Wnt, and Hippo pathways. The dysregulation of these pathways is linked to the fundamental hallmarks of BrCSCs, such as self-renewal, cancer resistance, metastasis, and recurrence [140, 141]. In addition, components in the tumor microenvironment, including hypoxia, angiogenesis, and inflammatory responses, have a main role in modulating BrCSCs [142, 143]. The Hh pathway components (PTCH1, Gli1, and Gli2) are abundantly expressed in normal mammary stem/progenitor cells and stimulated in BrCSCs. For the self-renewal of human BrCSCs, the Hh pathway and Bmi-1 are found to play critical roles highlighting the relevance of the Hh pathway and Bmi-1 in the controlling CSCs, hence applying techniques focused on blocking both pathways provide a helpful therapeutic strategy [144]. Chemotherapy has been demonstrated to be ineffective against BrCSCs, and the stemness of CSCs can be preserved through the Hh pathway. Using MCF-7 cells from BC patients as a model, Miao and his team discovered that the Hh signaling pathway is involved in drug responsiveness [145]. The Hh ligand secreted by CSCs can control the response of cancer-associated fibroblasts (CAFs). Indeed, the CAFs provide elements that encourage CSC growth and self-renewal [146]. Thus, inhibiting this signaling system might be a unique treatment method for BC therapy. Furthermore, SMO protein, transmembrane receptors (PTCH 1), (GLI 1–3), and extracellular Hh ligands are components of this complicated signaling cascade. Also, the Hh ligands activate signal transduction events in the cell, which interact with PTCH. To activate the transcription factor and allow it entry into the cell nucleus, the Hh ligand binds to PTCH, causing changes in its physical conformation and removing the inhibition of SMO. This results in increased cell function, growth, and differentiation [147, 148]. According to recent data, there is a clear relationship between lncRNAs and the cellular proliferation of BrCSCs through different signaling pathways (Table 2). For instance, LINC00617 increases the fraction of a phenotypic stem cell CD44( +)/CD24(-) subpopulation, enhancing BC metastasis. LINC00617 promotes EMT via rising the SOX2 expression in BC cells [50, 149]. Furthermore, lncRNA XIST expression in BC patients is linked to a higher risk of brain metastases. Reduced XIST expression promotes tumor cell stemness via stimulating EMT transition and activating c-Met through moesin MSN-mediated protein stabilization. In mouse mammary glands, knocking down XIST promotes the formation of primary tumors and brain metastases [150]. In addition, there is a clear relationship between EMT and the cellular proliferation of tumor cells. lncRNA-Hh, a lncRNA related to the Hh pathway, has been shown to directly bind GAS1 (a hedgehog signaling enhancer) and promote the activities of SOX2 and OCT4 [59]. Furthermore, lncROPM is a marker for BC patients' malignant grade, stage, and poor prognosis. Studies on the function gain and loss of lncROPM have shown that it is necessary to regulate BrCSC characteristics in vitro and in vivo studies. Through directly binding to the 3'-UTR of PLA2G16 (Group XVI phospholipase A2), lncROPM controls the expression of PLA2G16 by increasing the mRNA stability. Higher PLA2G16 enhances phospholipid metabolism and the generation of free fatty acids [151], particularly arachidonic acid, in BrCSCs, promoting PI3K/AKT, Wnt/-catenin, and Hippo/YAP pathways and ultimately contributing to the regulation of BrCSC stemness [152]. An animal investigation in BrCSCs found that H19 increased stemness via sponging Let-7c and activating its ESR1 and Wnt pathway targets [153]. Additionally, inhibition of symmetric division of BrCSCs by H19 or Let-7c miRNA increases non-CSCs. lncRNA-Hh is linked with the Shh-GLI1 pathway and is transcriptionally controlled by Twist, directly targets GAS1 to enhance Hh signaling activation. Increased GLI1 expression and increased SOX2 and OCT4 transcription are all associated with the activation of Hh, which is thought to play a regulatory function in CSC development [154]. Additionally, lncRNA-Hh stimulates the Hh pathway protein Hh to increase OCT4 and SOX2 expression for BrCSC homeostasis [59]. Similarly, Han et al. showed that lncRNA HOTTIP modulates the miR-148a-3p/WNT1 axis, which allows BrCSCs retain their CSC-like features and promote BC development [130]. In contrast, overexpression of LINC00968 in BC cells reduces the development of resistance by decreasing the stimulation of the Wnt2-β-catenin pathway by suppressing Wnt2 [155] (Fig. 4).

Altogether, these data imply that the lncRNAs (lncRNA-Hh, H19, LINC00617, lncROPM, XIST, PLA2G16) play a critical role in the regulation of CSCs and are associated with the invasive and migration characteristics of BC cells. They showed that these molecules might be useful in the prognostic and diagnostic method in metastatic BC patients.

## LncRNAs involved in cancer stemness and therapeutic resistance

Resistance to therapy is a hallmark of aggressive tumors, which leads to reduced survival and a high mortality rate. Patients with advanced or metastatic BC are often treated with chemotherapy, but some of patients acquire resistance to subsequent treatments [156]. Beside chemotherapy, hormonal therapies like tamoxifen are usually given in BC patients with estrogen-positive tumors. Loss of ER altered expression of co-regulatory proteins (CRPs), as well as increases in the number of ER-negative CSCs in the ER^+^ tumor, and lncRNAs mediates and contributes to tamoxifen resistance in BC [157–159].

Although cancer drugs effectively eliminate or kill the majority of cancerous cells, CSCs are able to evade their effects via a variety of biological strategies [160]. CSCs with slow division or quiescence, for example, are less affected by chemotherapy that targets rapidly dividing cells. Another critical resistance mechanism is aldehyde dehydrogenase (ALDH) overexpression in CSCs [161]. Enzymes in this family catalyze the oxidation of aldehydes to carboxylic acids, which is a step in the detoxification process for both internal and external aldehydes [162].

Induced iPSCs were shown to retain their pluripotency via lncRNA Peblr20, which suggests that lncRNAs may play a role in retaining cancer stemness [163]. EMT-associated stemness is controlled by lncRNAs, which have emerged as new participants in BrCSC stemness [124]. As an example, numerous lncRNAs, including LINC01638 [164], lncRNA RP1-506.5 [165], LINC-ZNF469-3 [166] and LINC-ROR [167, 168], have been shown to protect or promote EMT properties and CSC-properties of BC cells (Fig. 3). A recent study by Tang et al. found a direct link between lncRNA-regulated EMT and BC therapy resistance. According to their findings, DCST1-AS1 interacts effectively with ANXA1 to increase EMT and improve resistance in BC cells to doxorubicin and paclitaxel [169, 170].

In addition, it was determined that numerous pluripotency regulators, such as LIN28, SOX2, OCT3/4, KLF4, and CSC biomarkers, such as ALDH1A3, can stimulate stemness in BrCSCs [171–173]. By interacting with CSC-associated genes, lncRNAs play crucial role in BrCSC formation and can be used as a marker and applied for clinical translation. For instance, when CCAT2 lncRNA is expressed in breast cancer stem cells during targeted knockdown, it increases the levels of Nanog, OCT4, and KLF4, as well as the ALDH + CSC population which can serve as a marker for the presence cancer stem cells [174]. In particular, deregulation and circulating lncRNAs in physiological fluids of cancer patients may serve as useful diagnostic and prognostic markers in the treatment of the disease and can guide clinical therapy. For example, urine PCA3 (lncRNA PCA3) has been authorized by the FDA as a urine marker for prostate cancer due to its high sensitivity and specificity over PSA (prostate-specific antigen) [175, 176].

In addition, lncRNAs are reliable indicators of the patient's prognosis and the risk of their tumor recurrence. In clinical studies of breast cancer patients, hormone negativity, tumor size, and nodal status are all linked to high H19 expression. Disease-free survival is considerably worse in patients who has H19 expression than in other patients [66]. Furthermore, high expression of HOTAIR in BC patients is strongly correlated with lymph node metastasis, recurrent and poor prognosis of breast cancer [77, 177].

Likewise, many lncRNAs regulate the expression of pluripotency factors and CSC markers by functioning as ceRNAs that compete with the limited number of miRNAs (Fig. 3). According to Peng et al., the lncRNA H19 in BrCSCs acts as a sponge for Let-7, resulting in the increased expression of Let-7's target LIN28, which supports the maintenance of BrCSCs [67]. They found that LIN28 reduces Let-7 expression and activation, ultimately suppressing H19 transcription in BrCSCs. Treatment resistance may be restored by disruption of the H19-Let-7/LIN28 pathway, which is responsible in part for stemness of BrCSCs, and it presents a new strategy for treating BC. Similarly, the lncRNA LINC01133 induced by MSCs promotes BrCSC's morphological and growth features via directly regulating KLF4 [129].

NRAD1 (LINC00284) is notable for being the first lncRNA to be triggered by a CSC marker. In addition, it was shown that retinoic acid, a byproduct of ALDH1A3, has a beneficial effect on the expression of NRAD1, which promotes cell survival and increases BrCSCs [71]. Interestingly, the Knockdown of ALDH1A3 reduced the activity of ALDH, and ALDH1A1 knockdown suppressed metastatic features and therapy resistance in human BC cells [178].

While long noncoding RNAs play a significant role in maintaining BrCSCs and the potential development of intrinsic therapeutic resistance, further research into the underlying processes and potential clinical applications is needed.

## Challenges and strategies to overcome lncRNAs therapeutic resistance in BrCSCs

### Challenges to overcoming lncRNA therapeutic resistance

Despite advances in cancer treatment, drug resistance continues to be a significant challenge for people with BC. A comprehensive evaluation of off-target effects, possible toxicity, and drug delivery/precision targeting is necessary for the lncRNA silencing approaches to be considered effective therapeutic strategies. Many lncRNA transcript variations also present a considerable challenge for developing techniques to target molecules. It is possible that not all transcript variations will be targeted by the lncRNA silencing technique, which may reduce the treatment's effectiveness if that variant is functional. Moreover, CRISPR/Cas9 may have challenges or limited utility for the knockout of non-coding genes [179, 180]. Despite their equivalent molecular weight, there are no open reading frames (ORPs) in lncRNAs, unlike protein-coding genes. Furthermore, the roles of most lncRNAs remain unknown, making the creation of efficient medicines and delivery methods much more difficult. A comparison study in clinical trials would be required to determine whether or not lncRNAs are better targets than protein-coding genes [181].

### Strategies for reducing therapeutic resistance to lncRNAs

As previously stated, CSCs have responsibility for drug resistance and recurrence, all of which have an impact on anticancer therapeutic potential. The use of lncRNAs or related pathways to target CSCs as a possible therapy for cancer is a novel approach [182]. In BC, down-regulation of the oncogenic lncRNA CCAT1 is linked to an increase in radiosensitivity through altering the miR-148b, which is responsible for cancer progression [183]. Interestingly, Dendrosomal curcumin (DNC), a natural chemical, can be used to treat cancer by reducing the expression of tumor suppressor genes Tusc7 and GAS5 lncRNA in BC cells MCF7, MDA-MB231, and SKBR3. Furthermore, Esmatabadi and his team found that down-regulating GAS5 in BC cells can reduce many characteristics of DNC's anti-cancer activities. This suggests that combining DNC with GAS5 over-expression could be a clinically effective tool for drug-resistance in BC cells [184].

Recent study reveals that MEG3 interacts directly with miRNA-421 to regulate a variety of CSC characteristics, including self-renewal and invading abilities [185]. However, as a result of the methylation that occurs in its promoter region, the expression of the tumor suppressor lncRNA MEG3 is downregulated in cancer cells. A new nanotechnology-based preparation of natural curcumin known as dendrosomal curcumin increased the expression of MEG3 by down-regulating DNA methyltransferase (1A, 3A, and 3B) expression through promoting of miR-29a and miR-185 [186].

Chemoprevention is the focus of new strategies to enhance clinical outcomes and reduce the toxicity of anticancer medications. In the case of advanced ovarian cancer and BC, for example, Oxaliplatin (Oxa) is a platinum medication of the third generation that is used either alone or in combination with other treatments [187, 188]. However, several mechanisms contribute to resistance to Oxa, including DNA damage repair, inhibition of apoptosis, deregulation of signaling pathways, and increased detoxifying efficiency. Combination therapy has been proposed as an emerging approach to solving this problem [189]. It was found that DNC alone or in combination with Oxa has synergistically downregulated several oncogenic lncRNAs such as GAS5, MALAT1, FAL1, ANRIL, ABO73614, CCAT2, LSINCT5 in different types of cancers such as BC, NSCLC, and ovarian cancer through suppressing cell proliferation, prompting cell death, and reducing therapeutic resistance [184, 188, 190, 191]. They also found that DNC or curcumin combined with anti-cancer drugs had a more significant inhibitory effect than monotherapy. Curcumin reduces the lncRNA H19-induced EMT [192].

In addition, CRISPR interference (CRISPRi) or knockout (KO), a technology that may be applied to any genomic site, can be used to inhibit the activity of lncRNAs, which are crucial for the survival or self-renewal of cancer cells [193]. Complete excision of the whole gene, excision of the promoter and transcriptional start point, removing exon/exon junctions, or excision of the transcriptional terminal site are all strategies for lncRNAs knocking out [193]. For instance, CRISPR/Cas9 technology was used by Peng and colleagues to modify LncROR expression in BC cell lines. Researchers found that lncROR increased estrogen production through this new approach and triggered the MAPK/ERK axis in BC. CRISPR/cas9 technique was used in this study to investigate lncRNA loss of function and gives evidence for lncROR might be a possible target in ER^+^ BC patients [194]. Thus, it is a promising technique to reduce drug resistance, such as tamoxifen resistance of ER^+^ BC, and can be used as a therapeutic strategy in BC patients. In addition, lncRNA BC200 functions as an oncogene and has an important role in cancer proliferation and drug resistance [195]. Singh and colleagues published a notable study elucidating that oncogenic LncBC200 is elevated in BC [196]. The amount of LncBC200 in breast tumor tissues is greater in ER^+^ tumors than in ER^−^ tumors. They used CRISPR/Cas9 technology to knock off LncBC200 expression to understand more about the function of ER-regulated LncBC200 expression. According to the findings LncBC200 was shown to have a significant role in the development of cancer. Ultimately, oncogenic lncAK023948 which is essential for cancer cell survival has been used as a target for CRISPR/Cas9 to reduce tumor growth in different types of BC cells [197]. These findings offer a scientific basis for the concept that lncRNAs play an important part in the progression of cancer and could be used as therapeutic targets.

## Conclusions

It is predicted that therapies targeting CSC will prevent cancer metastasis and recurrence, since these cells have unique features that enhance anti-cancer therapy resistance. For this reason, identifying molecules that control their function is important in practice. The activity and growth of CSCs have been demonstrated to be influenced by lncRNAs. The effect of these regulatory transcripts on CSCs were mainly studied in BC. The activity and growth of CSCs were demonstrated to be influenced by lncRNAs. The effect of these regulatory transcripts on CSCs has mainly been studied in BC. LncRNAs may help BC patients not only as diagnostic indicators for identifying patient stage and prognosis but also as targets for precision treating cancer, thereby offering new pathways for eliminating CSCs. However, because of the complexity of CSC biology and the interrelationships among its various subtypes and their various cellular activities and signaling processes in health and illness, it is challenging to develop effective therapies that specifically target these cells. LncRNA-based research will help us achieve a new knowledge of the physiology of cancer stem cells. Furthermore, new studies are necessary to uncover and establish the wide range of regulatory mechanisms and the complexity of pathways that lncRNAs may control. Despite its limitations, understanding CSC-lncRNA interactions may usher in a new era of cancer therapy associated with lower drug resistance and increased anti-metastatic efficacy, thereby improving patient prognosis.

## Data Availability

Not applicable.

## Abbreviations

lncRNAs: Long non-coding RNAs
CSCs: Cancer stem cells
BrCSCs: Breast Cancer stem cells
AML: Acute myeloid leukemia
OCT4: Octamer-binding transcription factor 4
Sox2: Sex determining region Y-box 2
KLF4: Kruppel Like Factor 4
RNA: Ribonucleic acid
iPSCs: Induced Pluripotent stem cells
ESCs: Embryonic stem cells
MALAT1: Metastasis-associated lung carcinoma transcript 1
HOTAIR: Homeobox antisense transcript antisense RNA
EMT: Epithelial-to-mesenchymal transition
NR2F1-AS1: NR2F1 Antisense RNA 1
Xist: X-inactive specific transcript
MT1JP: Metallothionein 1 J
RNPs: Ribonucleoproteins
HRE: Hypoxia response element
HIF: Hypoxia-inducible factors
CAFs: Cancer-associated fibroblasts
PLA2G1: Phospholipase A2, group XVI
Hh: Hedgehog signaling
ER: Estrogen receptor
CRPs: Co-regulatory proteins
ORPs: Open reading frames
Oxa: Oxaliplatin
CRISPRi: CRISPR interference
KO: Knockout
CD 44: Transmembrane adhesion glycoprotein
